# Tumour necrosis factor-alpha inhibitors decrease mortality in COVID-19: a systematic review and meta-analysis

**DOI:** 10.1186/s13054-025-05420-9

**Published:** 2025-06-06

**Authors:** Ágoston Jánosi, Blanka Bódy, Rita Nagy, Klementina Ocskay, Tamás Kói, Katalin Müller, Ibolya Túri, Miklós Garami, Péter Hegyi, Andrea Párniczky

**Affiliations:** 1 Centre for Translational Medicine, Semmelweis University, Budapest, Hungary; 2 Heim Pál National Paediatric Institute, Budapest, Hungary; 3 Pharmaceutical Sciences and Health Technologies Division, Doctoral School, Semmelweis University, Budapest, Hungary; 4 Department of Stochastics, Institute of Mathematics, Budapest University of Technology and Economics, Budapest, Hungary; 5 Institute for Translational Medicine, Medical School, University of Pécs, Pécs, Hungary; 6 Department of Family Care Methodology, Faculty of Health Sciences, Semmelweis University, Budapest, Hungary; 7 András Pető Faculty, Semmelweis University, Budapest, Hungary; 8 Paediatric Centre, Semmelweis University, Budapest, Hungary; 9 Institute of Pancreatic Diseases, Semmelweis University, Budapest, Hungary

## Abstract

**Background:**

Despite widespread vaccination efforts, effective treatment strategies remain critical for severe SARS-CoV-2 infection. Tumour necrosis factor-alpha (TNF-α) plays a central role in the cytokine storm characteristic of severe COVID-19. This systematic review and meta-analysis evaluates the effectiveness, efficacy, and safety of TNF-α inhibitors in the management of COVID-19.

**Patients and methods:**

A systematic review of PubMed, Embase, and CENTRAL was conducted, focusing on studies involving SARS-CoV-2-infected patients treated with TNF-α inhibitors compared with those receiving standard of care without prior TNF-α inhibitor use. Data from studies published up to August 12, 2024, were analysed. Outcomes assessed included mortality, invasive mechanical ventilation, and C-reactive protein (CRP) levels. Odds ratios (ORs) and mean differences (MD) were calculated with 95% confidence intervals (CI), and subgroup analyses were performed for randomised controlled trials (RCTs) and non-randomised studies.

**Results:**

Seven studies involving 1393 patients with moderate-to-critical COVID-19 were included. TNF-α inhibitor treatment was associated with a reduced odds of mortality (OR 0.67, 95% CI [0.44–1.00], *P* = 0.052), which was statistically significant in the RCT subgroup across three studies (OR 0.75, 95% CI [0.58–0.97], *P* = 0.042, certainty of evidence: very low). The number needed to treat for mortality was calculated to be 16 (95% CI 9.0-inf.), which indicates that one additional death could be avoided for every 16 patients treated with TNF-α inhibitors compared to standard of care. No significant reduction in the need for invasive mechanical ventilation was observed (OR 0.95 [95% CI 0.46–1.94]; *P* = 0.822). Additionally, TNF-α inhibitors resulted in a significant reduction in CRP levels (MD − 21.9 mg/L [95% CI − 38.46 to − 5.34]; *P* = 0.024) within three to seven days post-treatment.

**Conclusion:**

Our study indicates a potential role for TNF-α inhibition in the treatment of COVID-19 as their use was associated with reduced mortality, but further studies are needed to provide robust evidence.

**Supplementary Information:**

The online version contains supplementary material available at 10.1186/s13054-025-05420-9.

## Introduction

Despite widespread efforts to control the spread of severe acute respiratory syndrome coronavirus 2 (SARS‑CoV‑2), managing coronavirus disease 2019 (COVID-19) cases which require hospital admission remains an ongoing challenge. Although existing therapeutic guidelines have undoubtedly contributed to mitigating the impact of the disease, their efficacy in addressing severe manifestations is limited [1]. Therefore, there is a critical need to explore alternative treatment options to manage severe and critical cases of COVID-19 more efficiently. One promising area of investigation focuses on the exaggerated inflammatory response seen in severe cases of COVID-19, often described as a “cytokine storm” [1]. This dysregulated immune reaction—marked by the excessive release of pro-inflammatory cytokines—has been closely linked to disease progression and poor clinical outcomes [2, 3]. Given the central role of the inflammatory cascade in COVID-19 pathogenesis, there is growing interest in the potential use of tumour necrosis factor-alpha (TNF-α) inhibitors as a therapeutic strategy to mitigate this response and improve patient outcomes [4, 5].

TNF-α inhibitors are well-established treatments for a range of severe immunoinflammatory diseases and have demonstrated substantial clinical benefit in conditions such as rheumatoid arthritis and inflammatory bowel disease (IBD) [4–6]. These agents—including infliximab, etanercept, adalimumab, certolizumab pegol, and golimumab—have significantly advanced the management of chronic inflammatory conditions, supported by robust evidence of their safety and efficacy [7–9]. In the context of the COVID-19 pandemic, the urgent need to control inflammatory damage has led to early studies exploring the potential of TNF-α inhibitors to reduce disease severity in COVID-19 [1, 4, 7, 10–12].

In the pathophysiology of COVID-19, the initial viral replication phase is typically followed by a robust inflammatory cascade, in which tumour necrosis factor-alpha (TNF-α) plays a central role [13–15]. Elevated TNF-α levels have been associated with adverse outcomes, including disease progression to the severe or critical stages [1, 16–18]. Consequently, TNF-α inhibitors hold promise as a therapeutic strategy to modulate the inflammatory response and potentially improve clinical outcomes in severe COVID-19 cases [2]. However, current evidence on the use of TNF-α inhibitors in COVID-19 remains limited and inconclusive, with studies reporting conflicting results.

Therefore, the primary objective of this study was to comprehensively summarise the available evidence on the potential effectiveness and safety of TNF-α inhibitors in the treatment of moderate-to-critical COVID-19 in inhibitor-naïve patients.

## Materials and methods

This meta-analysis was conducted in accordance with Cochrane recommendations and reported following the Preferred Reporting Items for Systematic Reviews and Meta-Analyses (PRISMA) statement [19, 20]. The protocol is registered in the International Prospective Register of Systematic Reviews (CRD42022286006).

### Data sources and search strategy

A comprehensive search was conducted on PubMed, Embase, and the Cochrane Register of Controlled Trials (CENTRAL) dated until August 12, 2024. The search query consisted of domains of COVID-19 and terms related to TNF-α inhibitors (Supplementary Material 1). Forward and backward citation searching was employed, using CitationChaser and Rayyan platforms [21, 22].

### Inclusion and exclusion criteria

Our meta-analysis investigated TNF-α inhibitor-naïve SARS-CoV-2 infected patients who received standard of care treatment with or without TNF-α inhibitors. Studies enrolling patients who had received TNF-α inhibitor therapy for non-COVID-19-related medical conditions were excluded, as were studies with fewer than ten SARS-CoV-2 infected participants. Eligible studies included randomised controlled trials (RCTs) and observational studies with control groups. There were no restrictions regarding language, diagnostic approach, disease severity, or geographic location. As a deviation from the protocol, the search period was extended, and no restrictions were imposed based on publication date. 

### Outcomes

Our primary objective was to analyse the mortality rate. Secondary outcomes included clinical improvement using the World Health Organisation (WHO) clinical progression scale, Intensive Care Unit (ICU) admission rates, length of ICU stay, length of hospital stay, and need for invasive mechanical ventilation [23]. Additional outcomes with comparable data from three or more articles in eligible studies were considered for discussion and meta-analysis. Ultimately, mortality, invasive mechanical ventilation requirement, and inflammatory response mitigation as indicated by C-reactive protein (CRP) levels were meta-analysed.

### Selection process and data extraction

Two independent reviewers, ÁJ and BB, used Rayyan.ai (Rayyan Systems Inc., 2020)—instead of Endnote as stated in the registered protocol for practical reasons—to perform the selection process by title-abstract and full text [22]. At each stage, the rate of agreement and Cohen’s Kappa were calculated (κ1: 0.95, κ2: 1) to assess selection quality, and any discrepancies were resolved via discussion. The WebPlotDigitizer online tool was used to extract data from the figures [24].

### Quality assessment

Two reviewers (ÁJ and BB) independently assessed the risk of bias, resolving discrepancies through discussion. The Revised Cochrane Risk of Bias Tool for Randomised Trials (RoB 2) was used for RCTs [25]. Deviating from our protocol, we employed the Joanna Briggs Institute (JBI) case–control tool instead of the preregistered Risk of Bias in Non-randomised Studies of Interventions (ROBINS-I) tool for non-randomised studies [26]. This decision was justified by better alignment with the included articles and enhanced suitability for risk of bias assessment. Quality and certainty of evidence were evaluated following recommendations from the Grading of Recommendations, Assessment, Development and Evaluation (GRADE) Working Group [27].

### Statistical analysis

Statistical analyses were conducted using R software (version 4.1.2), following the recommendations of Harrer et al. [28]. Odds ratios (OR) were calculated using the random-effect Mantel–Haenszel method (*metabin* function, *meta* R package), while mean differences (MD) were calculated to compare the decrease of CRP [between pre- and post-intervention means] using the random-effect inverse variance approach. The REML τ^2^ estimator was employed for between-study variance with Hartung-Knapp adjustment. Heterogeneity was assessed using I^2^ and the Cochrane Q test. RCTs and case–control studies were analysed as subgroups. The number needed to treat (NNT) was calculated from the pooled OR using the NNT function of the *meta* R package [29]. Contrary to our protocol, trial sequential analysis was omitted in accordance with the guidelines provided by the Cochrane Handbook [30]. For further information, please refer to Supplementary Material 2.

## Results

### Search and selection

**Fig. 1:**
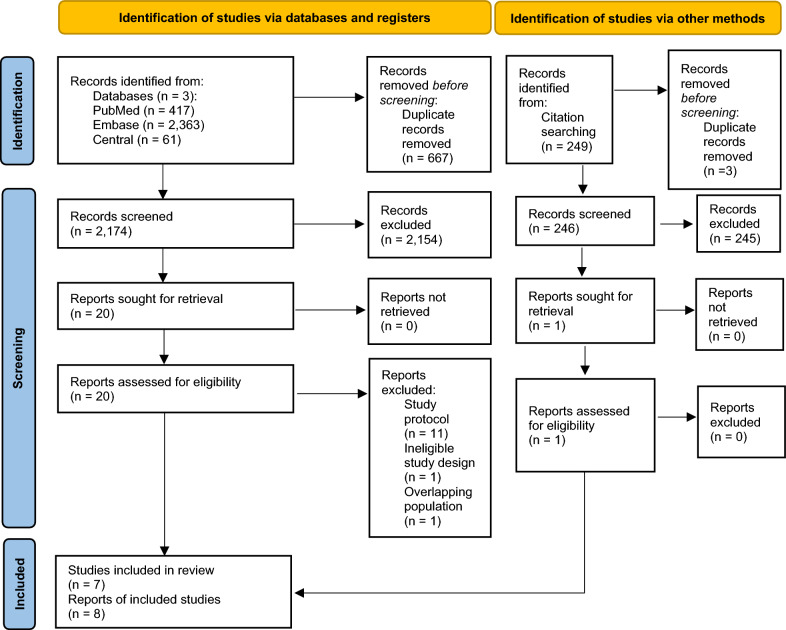
PRISMA flowchart of the selection process

The initial search identified 2,841 records, from which seven eligible articles were found (Fig. 1). These included three RCTs and three non-randomised case–control studies investigating the efficacy of TNF-α inhibitor treatment (infliximab or adalimumab) compared with the standard of care [31–37]. The included articles encompass the ACTIV-1 IM study, the outcomes of which have been reported in two separate publications [32, 33]. Additionally, forward citation chasing identified one more eligible study, resulting in a total of eight included articles (Supplementary Table 1) [38].

### Basic characteristics of included studies

The analysis involved 1,393 patients (all above the age of 16) with moderate-to-critical COVID-19, most of whom had radiological evidence of pulmonary involvement (Table 1). The mean patient age ranged from 53 to 72 years per study arm. For detailed eligibility criteria used in the included studies, refer to Supplementary Table 2. Patients were recruited from the United Kingdom, Germany, Iran, Egypt, United States and Latin America between 2020 and 2022. Length of follow-up varied considerably across studies ranging from 7 to 60 days in the included RCTs. Most patients were recruited from the RCT referred to as the ACTIV-1 IM study, which had the largest sample size and longest follow-up period [32, 33]. In total, 635 patients received infliximab, administered as a single-dose intravenous infusion of 5 mg/kg. Forty-three participants were treated with adalimumab (40 mg administered subcutaneously once), and 714 participants received standard of care without targeted immunomodulatory agents. Standard of care protocols included supportive care with corticosteroids and remdesivir. In one study by Farrokhpour et al., based on patient condition and national guidelines, patients received three or four drug regimens, including Oseltamivir, Hydroxychloroquine, Lopinavir/Ritonavir/Atazanavir, and Ribavirin/Sofosbuvir [36]. The 3-arm study by Sarhan et al. was excluded from the quantitative analysis and analysed separately as tocilizumab was part of the treatment protocol in both intervention groups (infliximab/tocilizumab and tocilizumab) [38].

**Table 1:** Main characteristics of the included studies

Study	Country	Original study type	Eligibility criteria	Follow up time (days)	Mean (SD)/median (IQR)* age (years)		Treatment	Population (n)		Outcomes analysed quantitatively
					Intervention group	Control group		Intervention group	Control group	
Fakharian [37]	Iran	RCT	Severe or critical COVID-19	7	53.2 (12.9)	56.1 (11.5)	Adalimumab	34	34	Mortality, ventilation, CRP
Fisher [35]	United Kingdom	RCT	Hospitalised with COVID-19 pneumonia and with a CRP ≧ 40 mg/L	28	55.4* (46.1–70.5)	64.5* (51.9–71.9)	Infliximab	29	34	Mortality, CRP
ACTIV-1 IM [32, 33]	North and Latin America	RCT	Moderate or severe COVID-19 pneumonia	60	54.7 (14.9)	54.9 (14.7)	Infliximab	517	516	Mortality, ventilation
Farokhnia [31]	Iran	Case–control	Severe COVID-19 pneumonia	NR	60.6 (2.2)	66 (2.4)	Adalimumab	9	9	Mortality, ventilation, CRP
Farrokhpour [36]	Iran	Case–control	Severe COVID-19, intubated, admitted to ICU	NR	63.4 (17.6)	72.4 (16.1)	Infliximab	27	43	Mortality
Reuken [34]	Germany	Case–control	Severe COVID-19	NR	59** (53–72)	66** (54–72)	Infliximab	19	38	Mortality, ventilation, CRP
Sarhan [38]	Egypt	Case–control	Moderate to severe COVID-19	Until discharge	60.1 (12.9)	59.6 (13.5)	Infliximab + Tocilizumab	43	40	Mortality, ventilation, CRP

*RCT* Randomised controlled trial, *Ventilation* Invasive mechanical ventilation, *CRP* Decrease of C-reactive protein level, *ICU* Intensive care unit, *NR* Not reported, *SD* Standard deviation, *IQR* Interquartile range

*Reported as median and IQR

**Reported as mean and IQR

### Main outcomes

#### Mortality

**Fig. 2:**
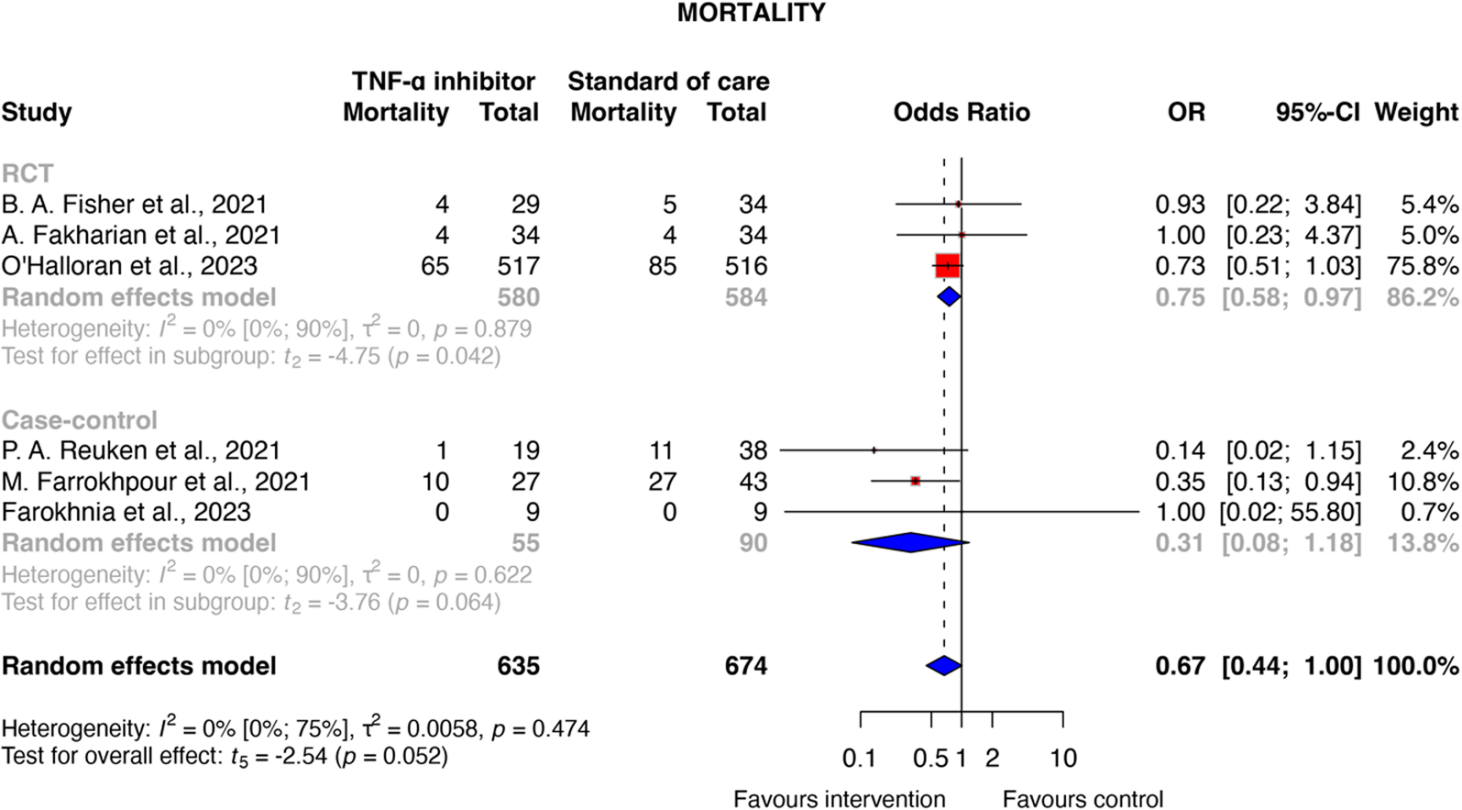
Odds ratio of mortality in COVID-19 patients with TNF-α inhibitor therapy compared to standard of care. *RCT* Randomised controlled trial, *OR* Odds ratio, *TNF* Tumour necrosis factor

Across the seven included studies, 236 deaths were reported (16.9%), 83 (35.17%) patients received infliximab, four patients (1.69%) received adalimumab, and 149 (63.13%) patients were in the standard of care group [31, 33–38]. Among the six articles included in the quantitative analysis, patients receiving TNF-α inhibitors exhibited a reduced odds of mortality compared to the control group (OR 0.67, 95% CI [0.44–1.00]; *P* = 0.052; see Fig. 2). Moreover, a subgroup analysis of RCTs affirmed these findings, demonstrating a statistically significant reduction in mortality (OR 0.75, 95% CI [0.58–0.97], *P* = 0.042). The calculated NNT ranged from 10 to 16 depending on baseline risk, meaning that between 10 and 16 patients must be treated with TNF-α inhibitors to prevent one additional death compared to the standard of care (Supplementary Material 3). One further study reported mortality, indicating that participants in the infliximab/tocilizumab group had better in-hospital survival compared to those who received tocilizumab and standard of care alone (*P* = 0.032) [38]. It should be noted that the level of certainty of evidence was very low for RCTs (Supplementary Material 4), and all studies—except for one RCT—were classified as carrying a high-risk of bias (Supplementary Material 5).

#### Clinical improvement

Clinical improvement based on the WHO ordinal scale has been reported by one study [35]. The median time for a 2-point improvement was 10 days (range 6–14) for the standard of care group and 15 days (range 6–21) for the infliximab group. In the ACTIV-1 IM trial, an 8-point ordinal scale was used. Overall, no statistically significant improvement was seen considering time to recovery (reaching ≥ 6 points on the ordinal scale) or clinical status at days 14 and 28 [33]. However, in-depth post-hoc analyses found that infliximab was associated with significant clinical benefit (HR 1.20, [95% CI 1.04, 1.39], *P* = 0.011) [32]. Sarhan et al. reported improvement in 79.1% of the participants in the intervention group, and 85.8% in the control group within 2 weeks, using a six-category clinical improvement scale [38]. Details in Supplementary Table 1.

### Additional outcomes

The need for mechanical ventilation and the biological effects of TNF-α inhibitors, particularly on CRP level reduction, were quantitatively assessed. Due to insufficient data for quantitative synthesis, ICU admission rates, length of ICU stay, and length of hospital stay were analysed qualitatively. All outcomes reported in the included studies are listed in Table 1 and Supplementary Tables 1 and 2.

#### Need for invasive mechanical ventilation

**Fig. 3:**
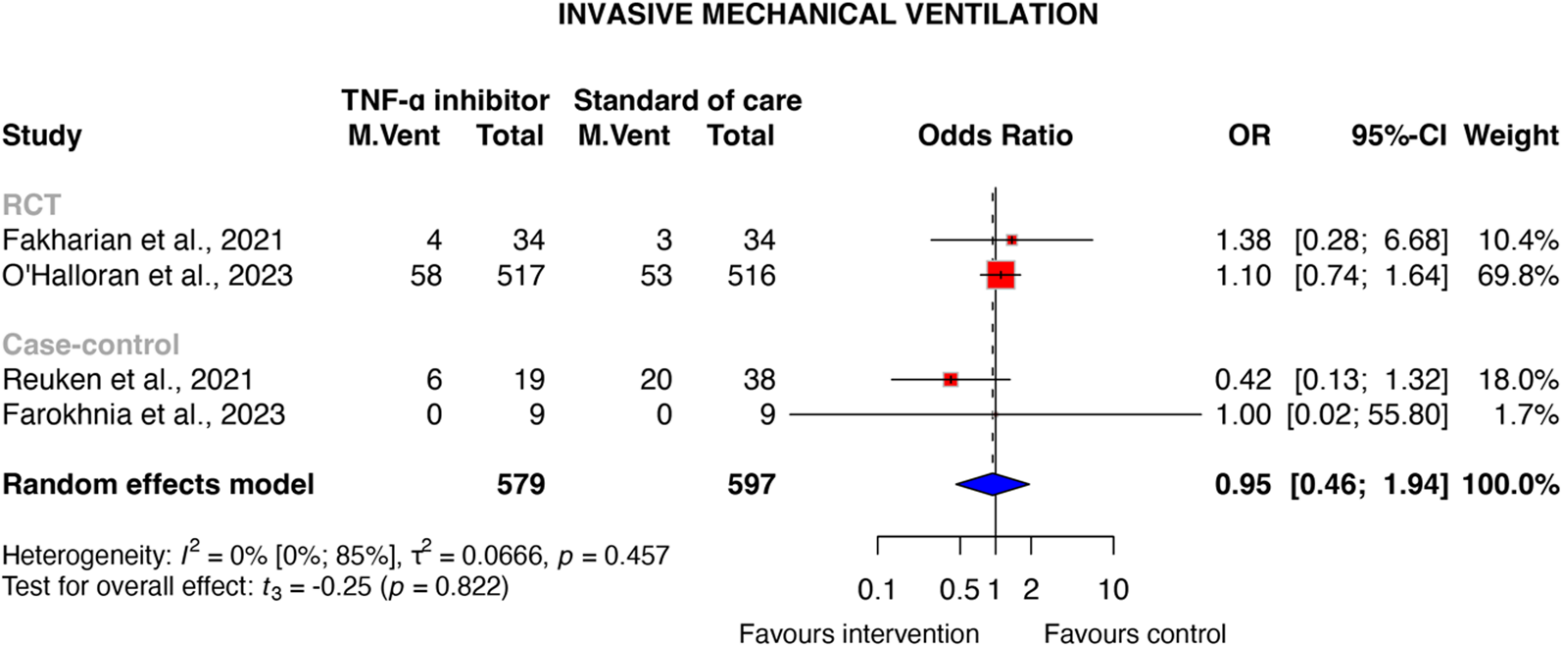
Need for invasive mechanical ventilation in COVID-19 patients with TNF-α inhibitor therapy compared to standard of care. *RCT* Randomised controlled trial, *OR* Odds ratio, *TNF* Tumour necrosis factor, *M. vent* Invasive mechanical ventilation

The odds of invasive mechanical ventilation were comparable among TNF-α inhibitor-treated participants and controls (OR, 0.95 [95% CI 0.46–1.94]; *P* = 0.822), with low statistical heterogeneity (*I*^2^: 0%, [95% CI 0–85%]). The analyses included two RCTs and two case–control studies with 1,176 participants (579 in the intervention group and 597 in the standard of care group) [31, 33, 34, 37]. Taking into account the limited data and wide prediction interval, caution is needed when interpreting these results (see Fig. 3). Qualitative analysis showed one patient (2.5%) in the intervention group and five patients (7%) in the control group requiring invasive mechanical ventilation [38]. All studies were considered to carry high risk of bias (Supplementary Material 5).

#### Intensive care unit admission

ICU admission was reported in three studies [35, 37, 38]. Fakharian et al. reported similar results in the adalimumab and standard of care groups with a 14.7% admission rate (*P* = 1) [37]. In the study by Fisher et al., 37% of patients in the infliximab group and 35% of those in the standard of care group received treatment in the ICU at the study’s outset [35]. In a study by Sarhan et al., 16 patients (37.2%) in the infliximab-tocilizumab group and 45 patients (65%) in the tocilizumab group required ICU admission [38].

#### Length of intensive care unit stay

Two of the included studies provided data on ICU length of stay [31, 37]. Fakharian et al. reported a median ICU stay of 13 days (range: 8–18.5 days) in the intervention group and 9 days (range: 6.5–19.5 days) in the control group (*P* = 0.53) [37]. In contrast, Farokhnia et al. observed an ICU stay of 4 days in the intervention group and 6 days in the control group (*P* = 0.5) [31].

#### Length of hospital stay

Three studies registered the length of hospital stay: Fakharian et al. reported a median length of 12.18 days in the adalimumab and 10.85 days in the standard of care group (*P* = 0.27; 95% CI − 1.08 to 3.73) [37]. Fisher et al. found that patients who received infliximab had a median hospital stay of 11 days (range: 2–28 days), whereas those in the standard of care group had a median stay of 10 days (range: 1–28 days) [35]. Sarhan et al. observed that patients in the intervention group had a length of hospital stay of 7.6 days, while those in the tocilizumab group had 8.9 days [38].

#### Safety and adverse events

Fisher et al. reported 102 adverse events in 69% of patients receiving infliximab and 112 events in 50% of patients receiving standard of care [35]. Furthermore, they observed six serious adverse events in the infliximab group and five in the standard of care group. However, all events were considered unrelated to treatment. O’Halloran et al. did not observe any differences in the composite safety end point on day 60 [33]. Serious adverse events were recorded in the cases of 125 participants in the intervention group and 130 participants in the standard of care group. Of these, six and seven events, respectively, were deemed treatment-related. The study conducted by Farokhnia et al. did not detect any short-term side effects associated with drug injection [31]. Sarhan et al. reported secondary bacterial infections (primarily sepsis) in 10 patients (22.5%) in the infliximab-tocilizumab group and in 12 patients (17.1%) in the tocilizumab group (Supplementary Table 1) [38].

#### Other outcomes

**Fig. 4:**
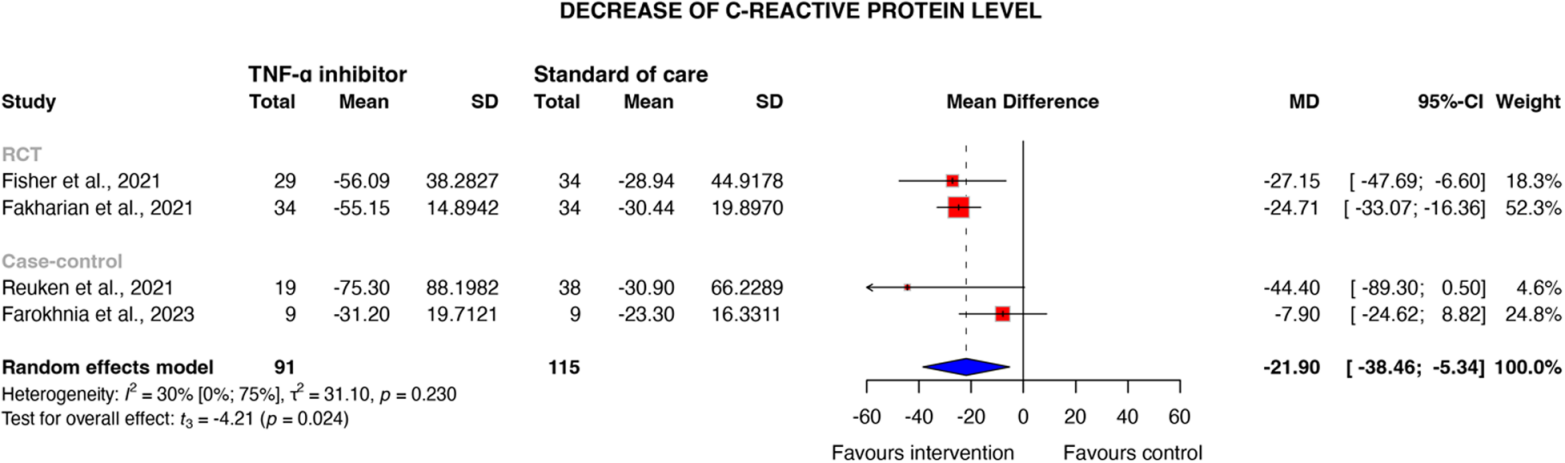
Decrease of C-reactive protein levels (mg/L) of COVID-19 patients treated with TNF-α inhibitor therapy compared to standard of care 3-to-7 days after treatment initiation, *MD* Mean difference, SD* Estimated standard deviation, *TNF* Tumour necrosis factor

In total, 5 studies reported CRP levels, allowing for the assessment of the biological effect of TNF-α inhibitors in COVID-19 [31, 34, 35, 37, 38]. Across four studies, patients treated with TNF-α inhibitors exhibited a significant reduction in CRP three to seven days after treatment initiation compared to controls (mean difference: − 21.9 mg/L [95% CI − 38.46 to − 5.34]; *P* = 0.024; see Fig. 4), indicating efficacy in intercepting inflammatory pathways [31, 34, 35, 37]. However, it is essential to acknowledge that all four studies had limited sample sizes, and variations in measurement timing may have influenced results. Additionally, Sarhan et al. reported significantly lower post-treatment CRP levels in the infliximab/tocilizumab group (*P* = 0.004) [38]. A high risk of bias was identified across all studies (Supplementary Material 5).

## Discussion

This study is the first to systematically evaluate the efficacy, effectiveness, and safety of TNF-α inhibitors in SARS-CoV-2 infected patients without prior immunomodulatory treatment.

While TNF-α inhibitors have not shown definitive efficacy in reducing mortality when considering both RCTs and non-randomised studies, the RCTs alone demonstrated a statistically significant reduction in mortality among patients with moderate-to-critical COVID-19 treated with TNF-α inhibitor therapy. In the context of published NNT-s for survival in severe or critical COVID-19, our findings suggest a relatively high efficacy of TNF-α inhibitors in preventing COVID-19-related death [39–41]. Further indirect support comes from observational studies of patients with autoimmune inflammatory diseases, where chronic TNF-α inhibitor therapy significantly reduced the odds of hospitalisation and severe COVID-19 outcomes (defined as ICU admission or death) [42, 43]. Adverse events associated with TNF-α inhibitor use were infrequent and comparable to those observed with standard care across the included studies, consistent with safety data from long-term use in other indications. These findings collectively underscore the potential applicability of TNF-α inhibitors in this patient population.

The use of TNF-α inhibitors in SARS-CoV-2 patients is in line with current understanding of the pathophysiology of severe COVID-19. Severe COVID-19 can be considered a form of viral sepsis, characterised by life-threatening organ dysfunction due to a dysregulated host response to SARS-CoV-2 infection [44]. Gharamti et al. have highlighted the importance of targeting pro-inflammatory cytokines in COVID-19, aligning with findings from Zawawi et al. and Jia et al., who identified elevated TNF-α levels as an independent risk factor for mortality in critically ill patients [2, 13, 44]. Despite the established link between elevated TNF-α levels and sepsis-related mortality, early trials of TNF-α inhibitors have yielded limited and often statistically insignificant therapeutic benefits [45, 46]. However, more recent meta-analyses have demonstrated a significant reduction in 28-day mortality with TNF-α blockade in sepsis [45, 47], reflecting the evolving understanding of cytokine modulation in critical illness and ongoing debate in sepsis research [44, 48].

Although our qualitative findings indicate a potential mortality benefit, it was based on limited data with high overall risk of bias, and clinical improvement could not be assessed quantitatively. Nonetheless, our study offers meaningful insight into the potential role of TNF-α inhibition in COVID-19 and underscores the urgent need for large, well-designed randomised trials to further clarify its therapeutic value and clinical implications.

### Strengths and limitations

Among the strengths, we emphasise the comprehensive nature of the study and the fact that it addresses a significant knowledge gap in the management of severe and critical COVID-19. To minimise potential bias, we included only studies enrolling patients without prior exposure immunomodulatory therapies and reported results separately for randomised controlled trials and case–control studies, the latter including two with matched controls.

Nonetheless, several limitations should be acknowledged. Data on clinically and economically relevant outcomes, such as length of hospital stay, were limited, and no paediatric studies were included. The overall sample size was relatively small, limiting the statistical power of the findings. Furthermore, heterogeneity in follow-up durations, severity classifications, and standard of care across studies, along with a generally high risk of bias, constrains the strength and generalisability of the available evidence.

### Implication for research

In light of our findings, further research is warranted to clarify the role of TNF-α inhibitors in COVID-19 treatment. Future RCTs should aim to determine the optimal timing of administration, identify patient subgroups most likely to benefit, and explore potential synergistic effects with other anti-inflammatory or antiviral agents. Moreover, long-term follow-up studies are needed to evaluate the durability of clinical benefits and to monitor for delayed adverse effects.

## Conclusion

Our study indicates a potential role for TNF-α inhibition in COVID-19 treatment; however, while TNF-α inhibitors were associated with reduced mortality, the current low-quality evidence does not establish their efficacy in improving clinical outcomes. These findings highlight the necessity for high-quality, large-scale studies to further evaluate their clinical impact in COVID-19.

## Supplementary Information

Additional file 1

## Data Availability

The datasets used and/or analysed during the current study are available from the corresponding author on reasonable request.
