# Supplementary material for: Tumour necrosis factor-alpha inhibitors decrease mortality in COVID-19: a systematic review and meta-analysis
Source: Crit Care. 2025 Jun 6;29:232. doi: 10.1186/s13054-025-05420-9 (PMC12144840; doi:10.1186/s13054-025-05420-9)
Supplement: Supplementary file 1 — Additional file 1 [file 13054_2025_5420_MOESM1_ESM.docx]

**SUPPLEMENT**

## ***Tumour Necrosis Factor-Alpha Inhibitors Decrease Mortality in COVID-19:***

## ***A Systematic Review and Meta-Analysis***

## Authors

Ágoston Jánosi, MD^1,2^; Blanka Bódy, MD^1,2^; Rita Nagy, MD, PhD^1,2^; Klementina Ocskay, MD, PhD^2,3^; Tamás Kói, PhD^1,4^; Katalin Müller, MD, PhD^1,2,5,6^; Ibolya Túri, PhD^1,7^; Miklós Garami, MD, PhD^1,8^; Péter Hegyi, MD, PhD, Dsc^1,5,9^; Andrea Párniczky, MD, PhD^1,2,5*^

## Affiliations

1. Centre for Translational Medicine, Semmelweis University, Budapest, Hungary

2. Heim Pál National Paediatric Institute, Budapest, Hungary

3. Pharmaceutical Sciences and Health Technologies Division, Doctoral School, Semmelweis University, Budapest, Hungary

4. Department of Stochastics, Institute of Mathematics, Budapest University of Technology and Economics, Budapest, Hungary

5. Institute for Translational Medicine, Medical School, University of Pécs, Pécs, Hungary

6. Department of Family Care Methodology, Faculty of Health Sciences, Semmelweis University, Budapest, Hungary

7. András Pető Faculty, Semmelweis University, Budapest, Hungary

8. Paediatric Centre, Semmelweis University, Budapest, Hungary

9. Institute of Pancreatic Diseases, Semmelweis University, Budapest, Hungary

*Correspondence: andrea.parniczky@gmail.com

## Corresponding author

Andrea Párniczky MD, PhD

Postal address: H-1089 Budapest, Üllői út 86., Hungary

E-mail address: andrea.parniczky@gmail.com

Table of contents

[1. Search key and data extraction 2](#_Toc174536295)

[2. Detailed statistical analysis 2](#_Toc174536296)

[Table 1. Systematic review 4](#_Toc174536297)

[Table 2. Eligibility criteria for included studies 13](#_Toc174536298)

[3. Number needed to treat analysis (random effects model) 17](#_Toc174536299)

[4. Certainty of evidence (Grade)^14^ 17](#_Toc174536300)

[5. Risk of bias assessment 19](#_Toc174536301)

[Bibliography 23](#_Toc174536302)

## 1. Search key and data extraction

| Concept 1 (population) | (covid-19 OR covid19 OR sars-cov-2) AND |
| --- | --- |
| Concept 2 (intervention) | (“anti tumor necrosis factor” OR “anti-tumor necrosis factor” OR “anti tumour necrosis factor” OR “anti-tumour necrosis factor” OR “tnf inhibition” OR “tnf-alpha inhibitor” OR “tnf alpha inhibitor” OR infliximab OR adalimumab OR etanercept OR certolizumab OR golimumab OR humira OR enbrel OR remicade OR simponi OR cimzia OR inflectra OR amjevita OR renflexis OR avsola OR ixifi OR hyrimoz OR hadlima OR erelzi OR cyltezo OR abrilada) |

We extracted the data into a standardised Microsoft Excel sheet, which included data regarding the article (first author, year of publication, digital object identifier, study design, and study duration), participants (baseline characteristics, laboratory parameters, and treatment applied per study group), outcomes and possible sources of bias.

## 2. Detailed statistical analysis

Statistical analyses were performed using the R statistical software (version 4.1.2.). The meta-analysis follows the advice of Harrer et al.^1^

For the odds ratio (OR) meta-analysis, we employed the random effects variant of the Mantel-Haenszel method, as implemented in the *metabin* function of the ‘*meta*‘ R package. For the continuous variable of C-reactive protein (CRP), we initially computed the pre- and post-intervention mean differences (MD) within both the intervention and control groups. Subsequently, we conducted a meta-analysis of the differences between these MDs using the classical random effects inverse variance method.

In all conducted meta-analyses, we utilised the REML τ^2 estimator. Given the limited number of contributing studies, the Hartung-Knapp adjustment was applied. Additionally, heterogeneity was evaluated by calculating the I² measure and its confidence interval, along with the Cochrane Q test. I² values ranging from 0% to 40% indicate potential unimportance, while those between 30% and 60% suggested moderate heterogeneity. Values within the range of 50% to 90% signify substantial heterogeneity, whereas values ranging from 75% to 100% indicate considerable heterogeneity.^2^

In instances where only the median and interquartile range of the continuous outcome were accessible, we applied the default approach of the *metacont* R function. Specifically, we estimated the mean and standard deviation utilising the methodologies delineated by Luo et al. and Wan et al.^3,4^

In each instance, despite having access to or being able to estimate the standard deviations of the outcome pre- and post-treatment, the standard deviation of the change was absent. In adherence to the guidelines outlined in the Cochrane Handbook, we substituted various correlations from the interval [0.3, 0.9]. Remarkably, all the utilised correlations yielded comparable outcomes. The reported findings were generated with a specified correlation of 0.7. Notably, study-specific confidence intervals varied significantly based on the correlation employed, whereas the aggregated outcome and its associated confidence interval remained relatively consistent. Subgroup analyses were conducted for randomised controlled trials and non-randomised studies. When a subgroup contained fewer than three studies, we visualised the study results without calculating the pooled estimate.

The number needed to treat (NNT) was computed from the combined odds ratio using the NNT function within the *meta* R package, in accordance with the guidelines outlined in the Cochrane Handbook.^2^ Given the dependence of NNT on baseline risk, we determined the NNT by considering the mean and maximum of the control event probabilities from the included studies as baseline risk, adhering to the default configuration of the specified function. Publication bias could not be assessed due to the small number of available studies.^5^

## Supplementary Table 1. Systematic review of all reported outcomes

| **Mortality** | | | |  | **Results** | | |
| --- | --- | --- | --- | --- | --- | --- | --- |
| **Study ID** | **Design** | **Intervention** | **Comparator** | **Outcome definition** | **Intervention** | **Comparator** | **P-value** |
| Fakharian et al, 2021^6^ | Randomised controlled trial | Adalimumab | Supportive care, Remdesivir, Dexamethasone | Rate of mortality until death or discharge  (number of patients) | 34/4 (11.7%) | 34/4 (11.7%) | 1.00 |
| Fisher et al, 2021^7^ (CATALYST) | Randomised controlled trial | Infliximab | Supportive care, Remdesivir, Dexamethasone, Tocilizumab for selected patients | Rate of mortality by day 28  (number of patients) | 29/4 (14%) | 29/5 (15%) | N/A |
| ACTIV1-IM (O'Halloran et al, 2023)^8^ | Randomised controlled trial | Infliximab | Supportive care, Remdesivir, Corticosteroids | ITT population:  Rate of mortality by day 14  (number of patients) | 531/29  (5.5%) | 530/42 (7.9%) | 0.0988 |
|  |  |  |  | ITT population:  Rate of mortality by day 28  (number of patients) | 531/53 (10.0%) | 530/75 (14.2%) | **0.0229** |
|  |  |  |  | ITT population:  Rate of mortality by day 60  (number of patients) | 517/65 (12.6%) | 516/85 (16.5%) | **0.0495** |
|  |  |  |  | TA population:  Rate of mortality by day 14  (number of patients) | 29/517  (5.6%) | 42/516 (8.1%) | 0.0923 |
|  |  |  |  | TA population:  Rate of mortality by day 28  (number of patients) | 52/517 (10.1%) | 75/516 (14.5%) | **0.0150** |
| Farokhnia et al, 2023^9^ | Case-control trial | Adalimumab | Supportive care, Remdesivir, Dexamethasone, Heparin | Mortality rate  (number of patients) | 9/0 | 9/0 | N/A |
| Farrokhpour et al, 2021^10^ | Case-control trial | Infliximab | Oseltamivir, Hydroxychloroquine, Lopinavir or Ritonavir or Sofosbuvir or Atazanavir and Ribavirin for selected patients | Death  (number of patients) | 27/10 (37%) | 43/27 (62.8%) | **0.05** |
| Reuken et al, 2021^11^ | Case-control trial | Infliximab | Supportive care, Remdesivir, Corticosteroids for selected patients | Rate of mortality by day 30  (number of patients) | 19/1 (5.2%) | 38/11 (28.9%) | N/A |
| Sarhan et al, 2023^12^ | Case-control trial | Tocilizumab + Infliximab | Supportive care, Dexamethasone, Tocilizumab, Enoxaparin, Remdesivir or Hydroxychloroquine or Lopinavir or Ritonavir | Death  (number of patients) | 43/3 (7%) | 70/10 (14.2%) | N/A |
| **Safety, adverse events** | | | | | **Results** | | |
| **Study ID** | **Design** | **Intervention** | **Comparator** | **Outcome definition** | **Intervention** | **Comparator** | **P-value** |
| Fisher et al, 2021^7^ (CATALYST) | Randomised controlled trial | Infliximab | Supportive care, Remdesivir, Dexamethasone, Tocilizumab for selected patients | Total AEs  (n, percentage of patients affected) | 102 (69%) | 112 (50%) | N/A |
|  |  |  |  | ≥Grade 3 AEs  (n, percentage of patients affected) | 79 (77%) | 102 (90%) | N/A |
|  |  |  |  | SAEs (n) | 6 (none related) | 5 | N/A |
|  |  |  |  | Infections | 4 in 4 patients | 7 in 4 patients | N/A |
| ACTIV1-IM (O'Halloran et al, 2023)^8^ | Randomised controlled trial | Infliximab | Supportive care, Remdesivir, Corticosteroids | Safety composite:  (deaths, SAEs, grade 3 or 4 AEs through day 60) (n) | 170 (32.9%) | 174 (33.7%) | N/A |
|  |  |  |  | SAEs  (death, a life-threatening AE, hospitalization or prolongation of existing hospitalization, persistent or significant incapacity or substantial disruption of the ability to conduct normal life functions, or a congenital anomaly/birth defect) (n) | 125 (24.2%) | 130 (25.2%) | N/A |
|  |  |  |  | SAEs related to study drug (n) | 6 (1.2%) | 7 (1.4%) | N/A |
|  |  |  |  | Grade 3 or 4 AEs (n) | 146 (28.2%) | 131 (25.4%) | N/A |
|  |  |  |  | Grade 4 AEs (n) | 63 (12.2%) | 64 (12.4%) | N/A |
|  |  |  |  | Grade 3 AEs (n) | 124 (24.0%) | 97 (18.8%) | N/A |
|  |  |  |  | AEs related to study drug (n) | 19 (3.7%) | 22 (4.3%) | N/A |
|  |  |  |  | Any secondary infections (n) | 79 (15.3%) | 72 (14.0%) | N/A |
|  |  |  |  | Confirmed secondary infections (n) | 24 (4.6%) | 26 (5.0%) | N/A |
|  |  |  |  | Probable secondary infections (n) | 55 (10.6%) | 46 (8.9%) | N/A |
| Farokhnia et al, 2023^9^ | Case-control trial | Adalimumab | Supportive care, Remdesivir, Dexamethasone, Heparin | Short-term side effects (n) | 0 | 0 | N/A |
| Sarhan et al, 2023^12^ | Case-control trial | Tocilizumab + Infliximab | Supportive care, Dexamethasone, Tocilizumab, Enoxaparin, Remdesivir or Hydroxychloroquine or Lopinavir or Ritonavir | Occurrence of myocarditis | 9.3% | 8.6% | N/A |
|  |  |  |  | Occurrence of myocardial infarction | 7% | 1.4% | N/A |
|  |  |  |  | Occurrence of heart failure | 2.3% | 2.9% | N/A |
|  |  |  |  | Occurrence of pulmonary embolism | 4.7% | 1.4% | N/A |
|  |  |  |  | Occurrence of hypertension | N/A | 1.4% | N/A |
|  |  |  |  | Occurrence of tachycardia | 0% | 2.9% | N/A |
|  |  |  |  | Occurrence of sepsis (n) | 10 (22.5%) | 12 (17.1%) | N/A |
| **Ventilation** | | | | | **Results** | | |
| **Study ID** | **Design** | **Intervention** | **Comparator** | **Outcome definition** | **Intervention** | **Comparator** | **P-value** |
| Fakharian et al, 2021^6^ | Randomised controlled trial | Adalimumab | Supportive care, Remdesivir, Dexamethasone | Mechanical ventilation requirement until death or discharge (n) | 4 (11.7%) | 3 (8.8%) | 1.00 |
|  |  |  |  | Nasal oxygen therapy (n) | 28 (82.3%) | 31 (91.1%) | 0.42 |
|  |  |  |  | Need for non-invasive ventilation (n) | 4 (11.7%) | 2 (5.8%) | 0.67 |
| Farokhnia et al, 2023^9^ | Case-control trial | Adalimumab | Supportive care, Remdesivir, Dexamethasone, Heparin | Face Mask (n) | 1 (11.1%) | 1 (11.1%) | 0.8 |
|  |  |  |  | Reservoir Bag Mask (n) | 7 (77.8%) | 6 (66.7%) | 0.8 |
|  |  |  |  | BIPAP (n) | 1 (11.1%) | 2 (22.2%) | 0.8 |
|  |  |  |  | Ventilator (n) | 0 | 0 | 0.8 |
|  |  |  |  | Number of Days Oxygen Needed  (mean ± SEM) | 7.2 ± 0.6 | 9.6 ± 1.8 | 0.06 |
| Farrokhpour et al, 2021^10^ | Case-control trial | Infliximab | Oseltamivir, Hydroxychloroquine, Lopinavir or Ritonavir or Sofosbuvir or Atazanavir and Ribavirin for selected patients | All included Cases intubated adults (n) | 27/27  (100%) | 43/43  (100%) | N/A |
| Reuken et al, 2021^11^ | Case-control trial | Infliximab | Supportive care, Remdesivir, Corticosteroids for selected patients | Ventilation (n) | 12 (66.6%) | 29 (79.2%) | N/A |
|  |  |  |  | Non-invasive ventilation (n) | 6 (33.3%) | 9 (23.7%) | N/A |
|  |  |  |  | Mechanical ventilation (n) | 6 (33.3%) | 20 (55.5%) | N/A |
| Sarhan et al, 2023^12^ | Case-control trial | Tocilizumab + Infliximab | Supportive care, Dexamethasone, Tocilizumab, Enoxaparin, Remdesivir or Hydroxychloroquine or Lopinavir or Ritonavir | Need for low oxygen (n) | 18.6% | 38.6% | N/A |
|  |  |  |  | Need for high oxygen / Non-invasive mechanical ventilation (n) | 69.8% | 14.3% | N/A |
|  |  |  |  | Need for invasive mechanical ventilation (n) | 1 (2.5%) | 5 (7%) | N/A |
| **Length of hospital stay** | | | | | **Results** | | |
| **Study ID** | **Design** | **Intervention** | **Comparator** | **Outcome definition** | **Intervention** | **Comparator** | **P-value** |
| Fakharian et al, 2021^6^ | Randomised controlled trial | Adalimumab | Supportive care, Remdesivir, Dexamethasone | Length of hospital stay  (days, median± SD) | 12.18 ± 4.64 | 10.85 ± 5.29 | 0.27 |
| Fisher et al, 2021^7^ (CATALYST) | Randomised controlled trial | Infliximab | Supportive care, Remdesivir, Dexamethasone, Tocilizumab for selected patients | Length of hospital stay  (days, median, range) | 11 (2-28) | 10 (1-28) | N/A |
| Farrokhpour et al, 2021^10^ | Case-control trial | Infliximab | Oseltamivir, Hydroxychloroquine, Lopinavir or Ritonavir or Sofosbuvir or Atazanavir and Ribavirin for selected patients | Length of hospital stay for survivors (days, mean ± SD) | 15.94 ± 7.66 | 13.81 ± 6.794 |  |
|  |  |  |  | Length of hospital stay for survivors (days, median, range) | 14 (7-37) | 13 (5-30) | 0.365 |
|  |  |  |  | Length of hospital stay for the deceased (days, mean ± SD) | 13.50 ± 8.33 | 7.44 ± 7.58 |  |
|  |  |  |  | Length of hospital stay for the deceased (days, median, range) | 13 (3-27) | 4 (1-28) | **0.021** |
| Sarhan et al, 2023^12^ | Case-control trial | Tocilizumab + Infliximab | Supportive care, Dexamethasone, Tocilizumab, Enoxaparin, Remdesivir or Hydroxychloroquine or Lopinavir or Ritonavir | Length of hospital stay  (days, mean) | 7.6 | 8.9 | N/A |
| **Length of ICU Stay** | | | | | **Results** | | |
| **Study ID** | **Design** | **Intervention** | **Comparator** | **Outcome definition** | **Intervention** | **Comparator** | **P-value** |
| Fakharian et al, 2021^6^ | Randomised controlled trial | Adalimumab | Supportive care, Remdesivir, Dexamethasone | ICU admission (n) | 5 (14.7%) | 5 (14.7%) | 1.00 |
|  |  |  |  | Length of ICU stay  (days, median, IQR) | 13 (8–18.5) | 9 (6.5–19.5) | 0.53 |
| Farokhnia et al, 2023^9^ | Case-control trial | Adalimumab | Supportive care, Remdesivir, Dexamethasone, Heparin | Length of ICU stay  (days, mean) | 4 | 6 | 0.5 |
| Farrokhpour et al, 2021^10^ | Case-control trial | Infliximab | Oseltamivir, Hydroxychloroquine, Lopinavir or Ritonavir or Sofosbuvir or Atazanavir and Ribavirin for selected patients | ICU admission duration for survivors  (days, mean ± SD) | 9.76 ± 5.22 | 5.62 ± 4.485 |  |
|  |  |  |  | ICU admission duration for survivors (days, median, range) | 7 (4-23) | 4.5 (1-17) | **0.006** |
|  |  |  |  | ICU admission duration for the deceased  (days, mean ± SD) | 9.10 ± 6.136 | 4.74 ± 4.16 |  |
|  |  |  |  | ICU admission duration for the deceased (days, median, range) | 7.5 (2-19) | 3 (1-15) | **0.031** |
| Sarhan et al, 2023^12^ | Case-control trial | Tocilizumab + Infliximab | Supportive care, Dexamethasone, Tocilizumab, Enoxaparin, Remdesivir or Hydroxychloroquine or Lopinavir or Ritonavir | ICU admission  (number of patients) | 16 (37.2%) | 45 (65%) | N/A |
| **Clinical improvement** | | | | | **Results** | | |
| **Study ID** | **Design** | **Intervention** | **Comparator** | **Outcome definition** | **Intervention** | **Comparator** | **P-value** |
| Fisher et al, 2021^7^ (CATALYST) | Randomised controlled trial | Infliximab | Supportive care, Remdesivir, Dexamethasone, Tocilizumab for selected patients | Time to 2-Point Improvement on WHO clinical progression scale  (days, median, 95% CI) | 15 (6–21) | 10 (6–14) | N/A |
| ACTIV1-IM (O'Halloran et al, 2023)^8^ | Randomised controlled trial | Infliximab | Supportive care, Remdesivir, Corticosteroids | ITT population:  Clinical status at day 14*  (OR, 95% CI) | 1.309 (1.041, 1.647) | | **-** |
|  |  |  |  | ITT population:  Clinical status at day 28*  (OR, 95% CI) | 1.435 (1.129, 1.825) | | **-** |
|  |  |  |  | ITT population:  Recovery** through day 28  (OR, 95% CI) | 1.122 (0.987, 1.275) | | 0.0793 |
|  |  |  |  | TA population:  Clinical status at day 14*  (OR, 95% CI) | 1.318 (1.047, 1.659) | | - |
|  |  |  |  | TA population:  Clinical status at day 28*  (OR, 95% CI) | 1.448 (1.136, 1.846) | | - |
|  |  |  |  | TA population:  Recovery** through day 28  (OR, 95% CI) | 1.130 (0.993, 1.286) | | 0.0631 |
| ACTIV1-IM  (Lin et al, 2024)^13^ |  |  |  | Estimates (95% CI) for the hazard ratios of improvement from baseline clinical status | 1.18 (1.04 1.39) | | - |
|  |  |  |  | Estimates (95% CI) for the hazard ratios of deterioration from baseline clinical status | 0.76 (0.59, 0.98) | | - |
|  |  |  |  | Estimates (95% CI) for the hazard ratios of overall benefit | 1.20 (1.04, 1.39) | | **0.011** |
| Sarhan et al, 2023^12^ | Case-control trial | Tocilizumab + Infliximab | Supportive care, Dexamethasone, Tocilizumab, Enoxaparin, Remdesivir or Hydroxychloroquine or Lopinavir or Ritonavir | Clinical improvement on a 6-category scale in two weeks  (number of patients) | 34 (79.1%) | 60 (85.8%) | N/A |
| **C-reactive protein level** | | | | | **Results** | | |
| **Study ID** | **Design** | **Intervention** | **Comparator** | **Outcome definition** | **Intervention** | **Comparator** | **P-value** |
| Fakharian et al, 2021^6^ | Randomised controlled trial | Adalimumab | Supportive care, Remdesivir, Dexamethasone | Before treatment  (mg/L, mean ± SD) | 70 (54–81) | 56 (38–73) | 0.053 |
|  |  |  |  | 3 days after treatment  (mg/L, mean ± SD) | 12 (4.75–22.25) | 22 (11–42) | **0.025** |
| Farokhnia et al, 2023^9^ | Case-control trial | Adalimumab | Supportive care, Remdesivir, Dexamethasone, Heparin | Admission  (mg/L, mean ± SEM) | 47.7 ± 9.1 | 40.6 ± 7.6 | 0.5 |
|  |  |  |  | Discharge  (mg/L, mean ± SEM) | 16.5 ± 5.4 | 17.3 ± 4.9 | 0.7 |
| Reuken et al, 2021^11^ | Case-control trial | Infliximab | Supportive care, Remdesivir, Corticosteroids for selected patients | On day 1  (mg/L, mean, 1^st^/3^rd^ quartile) | 165.6 (111.5; 267.1) | 151.7 (101.6; 217.7) | N/A |
|  |  |  |  | On day 7  (mg/L, mean, 1^st^/3^rd^ quartile) | 90.3 (61.9; 169.4) | 120.8 (56.8; 162.0) | N/A |
| **Others** | | | | | **Results** | | |
| **Study ID** | **Design** | **Intervention** | **Comparator** | **Outcome definition** | **Intervention** | **Comparator** | **P-value** |
| Fakharian et al, 2021^6^ | Randomised controlled trial | Adalimumab | Supportive care, Remdesivir, Dexamethasone | >50% improvement on CT scan  (number of patients) | 8 (23.5%) | 5 (14.7%) | 0.74 |
|  |  |  |  | Mean IL-6 value  before treatment  (pg/mL, mean ± SD) | 22.25 ± 12.01 | 16.1 ± 8.96 | **0.02** |
|  |  |  |  | Mean IL-6 value  3 days after treatment  (pg/mL, mean ± SD) | 17.8 ± 10.15 | 19.1 ± 11.51 | 0.71 |
|  |  |  |  | TNF-α  before treatment  (pg/mL, mean ± SD) | 6.5 (5.5–10.1) | 7.45 (6.02–10.45) | 0.68 |
|  |  |  |  | TNF-α,  3 days after treatment  (pg/mL, mean ± SD) | 6.9 (5.15–11.4) | 6.8 (5.9–10.2) | 0.91 |
|  |  |  |  | WBC  before treatment  (x10^3/µL, mean ± SD) | 7.7 (4.21–10.58) | 6.48 (4.62–9.96) | 0.82 |
|  |  |  |  | Mean WBC value  3 days after treatment  (x10^3/µL, mean ± SD) | 9.29 ± 3.79 | 10.8 ± 5.1 | 0.38 |
|  |  |  |  | D-Dimer  before treatment  (ng/mL, mean ± SD) | 806 (405–1490) | 460 (308–720) | 0.059 |
|  |  |  |  | D-Dimer  3 days after treatment  (ng/mL, mean ± SD) | 888 (435.5–1619) | 405 (264.25–1086.75) | **0.03** |
|  |  |  |  | Ferritin  before treatment  (ng/mL, mean ± SD) | 900.5 (463.75–1373.75) | 523 (295–1030) | 0.18 |
|  |  |  |  | Ferritin  3 days after treatment, mean  (ng/mL, mean ± SD) | 1273.88 ± 561.29 | 689.81 ± 567.91 | **0.005** |
| Farokhnia et al, 2023^9^ | Case-control trial | Adalimumab | Supportive care, Remdesivir, Dexamethasone, Heparin | LDH admission  (U/L , mean ± SEM) | 940.1 ± 126.6 | 651.6 ± 103.6 | 0.6 |
|  |  |  |  | LDH discharge  (U/L , mean ± SEM) | 732 ± 93.3 | 645.1 ± 114.6 | 0.7 |
|  |  |  |  | SpO2 before admission,  before extra oxygen  (%, mean ± SEM) | 80.44 ± 1.05 | 83.44 ± 1.2 | 0.5 |
|  |  |  |  | SpO2 discharge,  before extra oxygen  (%, mean ± SEM) | 89.2 ± 0.8 | 89.7 ± 0.6 | 0.3 |
|  |  |  |  | SpO2 admission,  after extra oxygen  (%, mean ± SEM) | 93.5 ± 0.2 | 94 ± 0.8 | 0.09 |
|  |  |  |  | SpO2 discharge,  after extra oxygen  (%, mean ± SEM) | 95.5 ± 0.2 | 95.3 ± 0.5 | 0.4 |
|  |  |  |  | Respiratory rate admission,  per minute  (n, mean ± SEM) | 27.7 ± 2.2 | 26.2 ± 0.8 | **0.02** |
|  |  |  |  | Respiratory rate discharge,  per minute  (n, mean ± SEM) | 18 ± 1.2 | 15.6 ± 0.7 | 0.8 |
| Reuken et al, 2021^11^ | Case-control trial | Infliximab | Supportive care, Remdesivir, Corticosteroids for selected patients | IL6  day 1  (ULN, mean, 1^st^/3^rd^ quartile) | 11.5 (8.5; 22.7) | 11.3 (5.6; 25.9) | N/A |
|  |  |  |  | IL6  day 7  (ULN, mean, 1^st^/3^rd^ quartile) | 4.7 (1.8; 9.9) | 4.1 (1.4; 12.1) | N/A |
|  |  |  |  | WBC  day 1  (/nL , mean, 1^st^/3^rd^ quartile) | 8.9 (5.9; 13.5) | 7.5 (5.0; 12.4) | N/A |
|  |  |  |  | WBC  day 7  (/nL , mean, 1^st^/3^rd^ quartile) | 7.0 (5.5; 10.0) | 7.2 (5.1; 11.9) | N/A |
|  |  |  |  | Lymphocytes  day 1  (/nL , mean, 1^st^/3^rd^ quartile) | 0.51 (0.39; 0.87) | 0.64 (0.41; 0.91) | N/A |
|  |  |  |  | Lymphocytes  day 7  (/nL , mean, 1^st^/3^rd^ quartile) | 0.83 (0.40; 1.32) | 0.62 (0.43; 1.13) | N/A |
|  |  |  |  | Ferritin  day 1  (µg/L, mean, 1^st^/3^rd^ quartile) | 2538 (1563; 2948) | 1695 (908; 2347) | N/A |
|  |  |  |  | Ferritin  day 7  (µg/L, mean, 1^st^/3^rd^ quartile) | 2294 (1269; 3758) | 1458 (933; 2589) | N/A |
|  |  |  |  | D-dimer  day 1  (µg/L, mean, 1^st^/3^rd^ quartile) | 543.0 (373.8; 745.8) | 437.0 (303.0; 2095.0) | N/A |
|  |  |  |  | D-dimer  day 7  (µg/L, mean, 1^st^/3^rd^ quartile) | 557.5 (330.8; 3750.1) | 460.0 (250.0; 1227.0) | N/A |
|  |  |  |  | Creatinine  day 1  (µmol/L, mean, 1^st^/3^rd^ quartile) | 70.7 (56.0; 102.3) | 88.0 (66.5; 147.5) | N/A |
|  |  |  |  | Creatinine  day 7  (µmol/L, mean, 1^st^/3^rd^ quartile) | 66.5 (55.3; 82.5) | 88.0 (58.0; 128.0) | N/A |
|  |  |  |  | Covid inflammation score value on day 1***  (mean, 1^st^/3^rd^ quartile) | 11 (10; 12) | 10 (9; 11) | N/A |
|  |  |  |  | Covid inflammation score value on day 7***  (mean, 1^st^/3^rd^ quartile) | 9 (7; 12) | 9 (7; 13) | N/A |

ITT: intention-to-treat, N/A: Not available, TA: treated-as-assigned, SAE: Serious adverse event, AE: Adverse event, WBC: white blood cell, SEM: standard error of the mean, n: number, CI: Confidence interval, SD: Standard deviation, ICU: Intensive care unit, WHO: World Health Organization, CT: Computed tomography, TNF-α: Tumour necrosis factor-alpha

*’Clinical status (8-point ordinal scale) at 14 and 28 days calculated as proportional odds model by ordinal logistic regression. A number greater than 1 favours Infliximab’^8^

**’Recovery rate ratio and p value calculated using a stratified Fine Grey model with death as a competing risk. A number greater than 1 favors infliximab’^8^

***COVID hyperinflammation score: ^14^

Points

| Chest-X-ray/Chest-CT consistent w/hypersensitivity pneumonitis | 3 |
| --- | --- |
| CRP > 20 × ULN | 2 |
| Ferritin > 2 × ULN | 2 |
| Triglycerides > 1.5 × ULN | 1 |
| IL-6 > 3 × ULN | 1 |
| Fibrinogen > ULN | 1 |
| Leukocytes > ULN | 1 |
| Lymphopenia < 1.1/nL | 2 |
| Fever > 38.5 °C | 2 |
| Coagulation disorder | 1 |
| - DIC (D-Dimer > ULN) |  |
| - PTT > ULN |  |

ULN: Upper limit of normal, DIC: Disseminated Intravascular Coagulation, PTT: Partial thromboplastin time, CRP: C-reactive protein

## Table 2. Eligibility criteria for included studies

|  | **Randomised controlled trials** | | | |
| --- | --- | --- | --- | --- |
| **Study** | **Eligibility criteria** | **Exclusion criteria** | **Standard of care** | **Reported outcomes** |
| Fakharian et al.^6^ | ‘Patients with severe illness at ages from 18 to 70 years old with COVID-19 were considered as the cases for study. The criteria for identifying the cases were Reverse Transcription-Polymerase Chain Reaction (RT-PCR) reports and Computed Tomography (CT) scan confirming bilateral pulmonary infiltration. Severe or critical cases of COVID-19 were determined based on the SpO2 ≤ 93% at room air, Heart rate ≥ 125 min, respiratory rate ≥ 30/min, The evidence of shock, the need for mechanical ventilation or vasopressors, clinically significant acute hepatic, renal or neurological dysfunction due to COVID-19 and patients with acute respiratory distress syndrome.’ | 'Denying to sign the consent form; acute or chronic kidney disease proven by a rise in serum creatinine by more than 0.3 mg/dl during 48 h or glomerular filtration rate lower than 30 ml/min; the history of liver failure (ChildPugh stage C and D or more than 5 times rise above the upper limit of normal in liver function tests or 3 times in patients with symptoms of liver failure); the history of malignancy; the history of heart failure; patients with latent or active tuberculosis or any active infection; patients receiving medications affecting IL-6 or TNF-α levels; patients with active peptic ulcer disease; patients with a history of an allergic reaction to adalimumab or developing allergic reaction while receiving the medication; mildly ill patients; and pregnancy or breastfeeding.' | Remdesivir, Dexamethasone, supportive care | Requiring invasive mechanical ventilation, the necessity of admission to the ICU, rate of mortality, length of stay in the ICU or hospital, improvement observed in the chest CT scan. |
| Fisher et al.^7^ | ‘Age 16 years or older, admitted to hospital with a clinical picture strongly suggestive of SARS-CoV-2 pneumonia (confirmed by chest x-ray or CT scan, with or without a positive RT-PCR assay), and with a C-reactive protein (CRP) concentration of 40 mg/L or greater. The requirement for raised CRP concentration replaced an inclusion criterion for low oxygenation status (oxygen saturation ≤94% while breathing ambient air or a ratio of partial pressure of oxygen to the fraction of inspired oxygen of ≤300 mmHg) early in the course of recruitment, following a change in primary outcome.’ | Not meet the eligibility criteria | Supportive care ± dexamethasone, remdesivir, tocilizumab (2 patients (6%) in the Infliximab, 1 patient (3%) in the standard of care group received tocilizumab) | CRP concentration, WHO clinical progression scale (time to 2-point improvement), hospital survival status, hospital-free days, length of hospital stay, proportion of patients discharged at day 28, SpO2:FiO2 ratio, adverse events. |
| ACTIV1-IM (O'Halloran et al, 2023, Lin et al 2024).^8,13^ | ‘Participants 18 years or older with confirmed SARS-CoV-2 infection within 14 days, anticipated hospital stay of 72 hours or more, and evidence of pulmonary involvement were eligible.’ | ‘Candidates with pregnancy, liver enzymes more than 10 times the normal value, chronic liver disease, acute kidney injury with glomerular filtration rate less than 30 mL/min (stable chronic kidney insufficiency permitted), severe heart failure, severe neutropenia, lymphopenia, or known or suspected untreated infection including tuberculosis and those who received cytotoxic or biologic-targeted immunomodulators within 4weeks or 5 half-lives before screening were excluded.’ | Standard care including remdesivir and corticosteroids | Median time to recovery by day 28 was evaluated using an 8-point ordinal scale, mortality at day 28, Clinical status at day 14, mortality at day 60, adverse event. |

|  | **Case-control studies** | | | |
| --- | --- | --- | --- | --- |
| **Study** | **Eligibility criteria** | **Exclusion criteria** | **Standard of care** | **Reported outcomes** |
| Farokhnia et al.^9^ | ‘Patients with COVID-19 (diagnosis based on positive Reverse Transcription-Polymerase Chain Reaction (RT-PCR) test) were admitted to the study, considering inclusion and exclusion criteria. Inclusion criteria include severe diseases (pneumonia with hypoxia), need for oxygen, and 60 - 70 years old. | ‘Exclusion criteria also include displeasure with participation in the study, fever > 40 degrees, any active bacterial or fungal infection, positive blood and urine culture, history of chronic obstructive pulmonary disease, history of tuberculosis, history of heart failure, history of malignancy, active skin ulcer, the history of multiple sclerosis, extremely ill (shock, encephalopathy, myocardial lesion, coagulation, and renal impairment), abnormal liver tests (liver enzymes more than 2 times normal), patients with a history of allergic reactions to adalimumab drug or other medications, and glomerular filtration rate (GFR) ≤ 30.’ | Dexamethasone, remdesivir, and heparin in addition to supportive therapies | Requiring mechanical ventilation, number of days needed for oxygen, length of stay in the ICU, and SpO2 level and RR at the time of admission and discharge, as well as recovery or mortality, CRP and LDH level on day 1 and 5. |
| Farrokhpour et al.^10^ | 'Patients over 18 years of age with severe COVID-19 infection (based on clinical symptoms and radiographic or CT scan parameters) who were intubated and admitted to the ICU. Cases with symptom of tachypnoea (respiratory rate > 35/min) and heart rate > 125 beat/min, and Spo 2 <85% on mask with reservoir, loss of consciousness, and unstable vital sign were intubated, and admitted to the ICU. All cases enrolled in the study had multilobar ground-glass opacities, and consolidations on chest computed tomographic (CT) scan.' | Not reported | Oseltamivir + hydroxychloroquine + lopinavir/ritonavir or sofosbuvir or atazanavir ± ribavirin | Discharge, death, hospitalisation duration, ICU admission duration, survival time. |
| Reuken et al.^11^ | Patients hospitalized for severe, PCR-proven COVID-19 (age and sex matched controls). | Not reported | Non-specific anti-inflammatory treatment ± corticosteroids (Infliximab group: 72.2%; standard of care group: 69.4%), remdesivir (infliximab group: 66.7%; standard of care group: 63.9%) | 30-day-Mortality, Ventilation (non-invasive, mechanical),  Laboratory parameters on day 1 and 7: IL-6, CRP, WBC, limphocytes, ferritin, D-Dimer, Creatinine, Covid inflammation score on day 1 and 7. |
| Sarhan et al.^12^ | ‘Patients over 18 who were hospitalized with pneumonia confirmed by chest CT scan and tested positive for COVID-19 infection using RT-PCR were included in the study. We included patients who had CSS which was detected by inflammatory markers elevation; either C-reactive protein (CRP) ≥ 100 mg/L or ferritin ≥ 900 ng/mL, along with lactate dehydrogenase (LDH) > 220 U/L and interleukin-6 level (IL-6) > 10 pg/ ml. In addition to one of the following: respiratory rate ≥ 30 respirations/min, oxygen saturation ≤ 93%, ratio of pressure arterial oxygen partial pressure to inspired oxygen fraction (PaO2/FiO2) < 300 or who showed worsening of pulmonary areas of consolidation, defined as increase in number and size of patches.’ | ‘Patients with evidence of concurrent bacterial infection, use of other immunosuppressant, and levels of alanine aminotransferase (ALT) or aspartate aminotransferase (AST) that are five times higher than the upper range of normal were excluded. Additionally, patients who received treatment with anti-TNFα in the previous month and show hypersensitivity to any TNFα inhibitor as well as active or latent tuberculosis were excluded. Patients who received any COVID-19 vaccine or those who were previously infected with SARS-COV were also excluded from the study.’ | ‘All patients received the standard treatment of 400 mg of hydroxychloroquine once daily, or 400/100 mg of lopinavir/ritonavir twice daily, or remdesivir 200 mg as a loading dose followed by 100 mg once daily as a maintenance dosage, in addition to dexamethasone 6 mg once daily for 7–10 days. Anticoagulant enoxaparin is administered subcutaneously once daily as a preventative measure if the D-dimer is between 500 and 1000, and twice daily as a therapeutic measure if the D-dimer is greater than 1000. Patients also received supportive treatments of quetiapine (25 mg once a day at bedtime) and paracetamol (1 g every 6 h).’ | The length of hospital stay, admission to the intensive care unit (ICU), death, the use of NIV or invasive mechanical ventilation, the development of secondary infections (such as bacterial or fungal infections), elevation in liver enzymes above three times the normal threshold. |

## 3. Number needed to treat analysis (random effects model)

|  | **OR** | **p-value**  **(p.c)** | **Number needed to treat for benefit (NNTB)** | **95%-CI** |
| --- | --- | --- | --- | --- |
| **Max** | 0.6672 | 0.2245 | 15.9711 | [NNTB 9.0252 to Inf to NNTH 1233.2802] |
| **Mean** | 0.6672 | 0.6279 | 10.1732 | [NNTB 4.9978 to Inf to NNTH 920.6320] |

OR: odds ratio, Inf: infinity, NNTH: number needed to treat harm

NNTB represents the number of patients who need to receive the treatment for one additional patient to benefit compared to the control group.

NNTH refers to the number of patients who need to be treated with the intervention for one additional patient to experience harm compared with the control group.

## 4. Certainty of evidence (Grade)^15^

As the certainty of evidence is greatly influenced by study design, we assessed the RCTs separately. The certainty of evidence for the primary outcome (mortality) was very low. The risk of bias was high in all three included studies, which seriously affected the assessment of certainty. We conclude that there were no significant inconsistencies in the results of these studies. The evidence answers to the healthcare questions were indirect. The three studies investigating the healthcare question used different observation times and TNF- α inhibitor medicines (infliximab, adalimumab). The results are imprecise due to wide confidence intervals overlapping with the null effect line.

| **Certainty assessment** | | | | | | **No. of patients** | | **Effect** | |  | | |
| --- | --- | --- | --- | --- | --- | --- | --- | --- | --- | --- | --- | --- |
| **Study design** | **Risk of bias** | **Inconsistency** | **Indirectness** | **Imprecision** | **Other considerations** | **anti-TNF alpha** | **Standard of care** | **Relative**  **(95% CI)** | **Absolut**  **(95% CI)** | | **Overall certainty of evidence** | |
| Randomised trials  No. 3. | serious* | not serious | serious** | serious*** | none | 73/580  (12.6%) | 94/584  (16.1%) | **OR: 0.78**  (0.63 to 0.97) | 35 fewer per 1000  (from 60 fewer to 5 fewer) | ⨁◯◯◯ VERY LOW | |  |

**Mortality**

CI: Confidence interval, OR: Odds ratio

Explanations:

* A high risk of bias is observed.

** Time differences and differences in the intervention (infliximab, adalimumab).

*** Wide confidence intervals. The 95% confidence interval overlapped with no effect.

## 5. Risk of bias assessment

Most studies included in the analysis were found to have a high risk of bias for the outcomes assessed. The study by Fakharian et al. was the only one rated as having a low risk of bias for mortality outcome.^6^

**1.) For randomised controlled trials, the Revised Cochrane Risk of Bias Tool for Randomized Trials (RoB 2) was used to assess the risk of bias.**^16^

| **Study ID** | **Outcome** | **D1: Randomisation process** | **D2: Deviations from the intended interventions** | **D3: Missing outcome data** | **D4: Measurement of the outcome** | **D5: Selection of the reported result** | **Overall** |  |  |  |
| --- | --- | --- | --- | --- | --- | --- | --- | --- | --- | --- |
| Fisher et al. | Mortality |  |  |  |  |  |  |  |  | Low risk |
| Fakharian et al. | Mortality |  |  |  |  |  |  |  |  | Some concerns |
| O'Halloran et al. | Mortality |  |  |  |  |  |  |  |  | High risk |
| Fakharian et al. | Ventilation |  |  |  |  |  |  |  |  |  |
| O'Halloran et al. | Ventilation |  |  |  |  |  |  |  |  |  |
| Fisher et al. | Decrease of CRP level |  |  |  |  |  |  |  |  |  |
| Fakharian et al. | Decrease of CRP level |  |  |  |  |  |  |  |  |  |

**2.) For non-randomised studies JBI case-control tool was used.**^17^

**1.     Were the groups comparable other than the presence of disease in cases or the absence of disease in controls?**

Controls were representative of the source population that produced cases. This was done through individual or group matching.

**2.     Were cases and controls matched appropriately?**

Clearly and carefully defined sources from which cases and controls were recruited.

**3.     Were the same criteria used for identification of cases and controls?**

Patients were included based on specified diagnoses, and definitions or patients were matched according to key characteristics. The cases and controls had the same eligibility criteria with clear definitions.

**4.     Was exposure measured in a standard, valid and reliable way?**

The measurement of exposure was appropriate and clearly defined.

**5.     Was exposure measured in the same way for cases and controls?**

The exposure measurement was clearly defined and assessed using the same procedure for both cases and controls.

**6.     Were confounding factors identified?**

Potential confounding factors (baseline characteristics, prognostic factors, and concomitant exposures) were identified and measured if it was possible.

**7.     Were strategies to deal with confounding factors stated?**

There was an appropriate strategy to address confounders (multivariate, logistic regression analysis).

**8.     Were outcomes assessed in a standard, valid and reliable way for cases and controls?**

The outcome definitions and measurements are clearly described. The measurement was appropriate, and the measurement tools were validated.

**9. Was the exposure period of interest long enough to be meaningful?**

The appropriate exposure period determines the duration of drug administration and follow-up. Reuken et al. did not provide any data on ventilation needs.^11^ Farrokhpour et al. included only ICU-admitted intubated patients.^10^

**10.  Was appropriate statistical analysis used?**

Described analytical techniques and measured confounders.


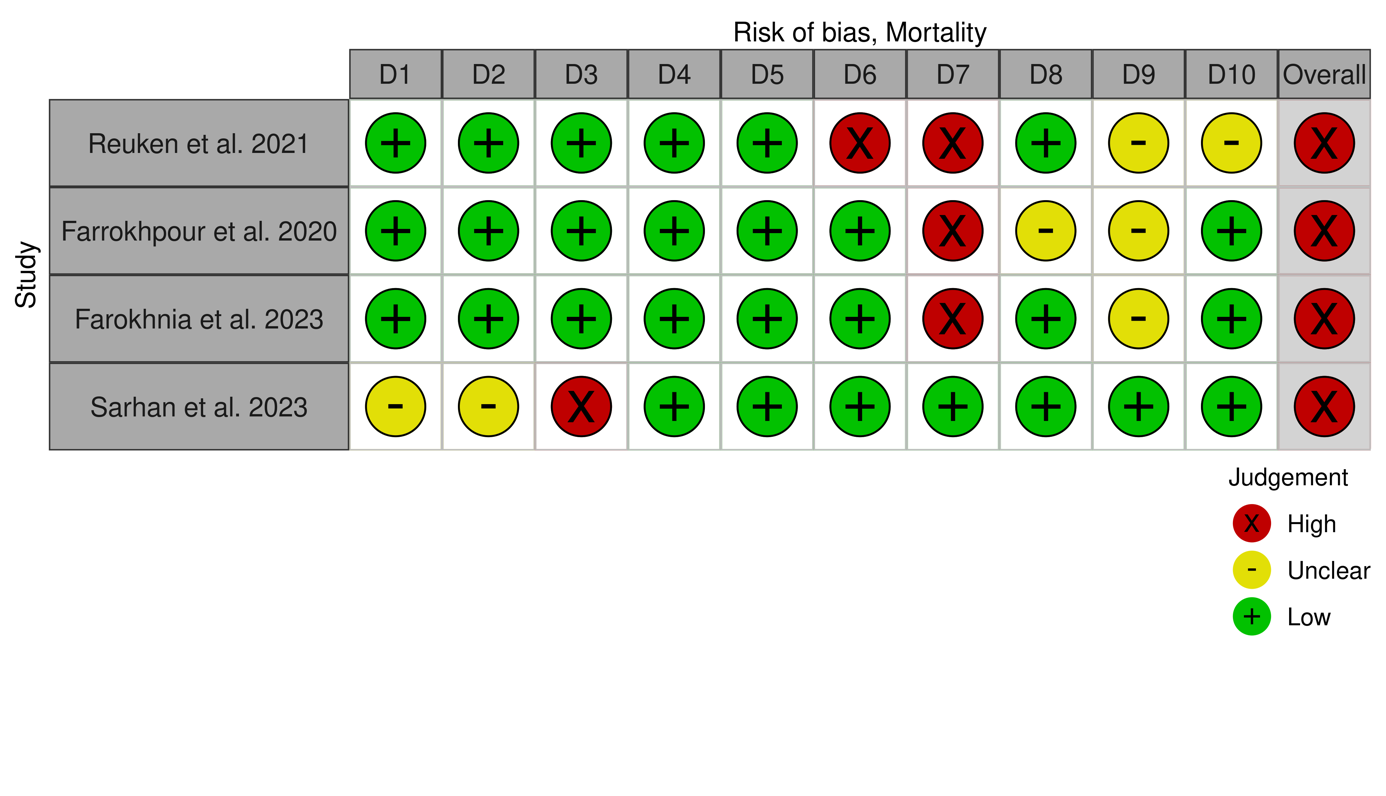


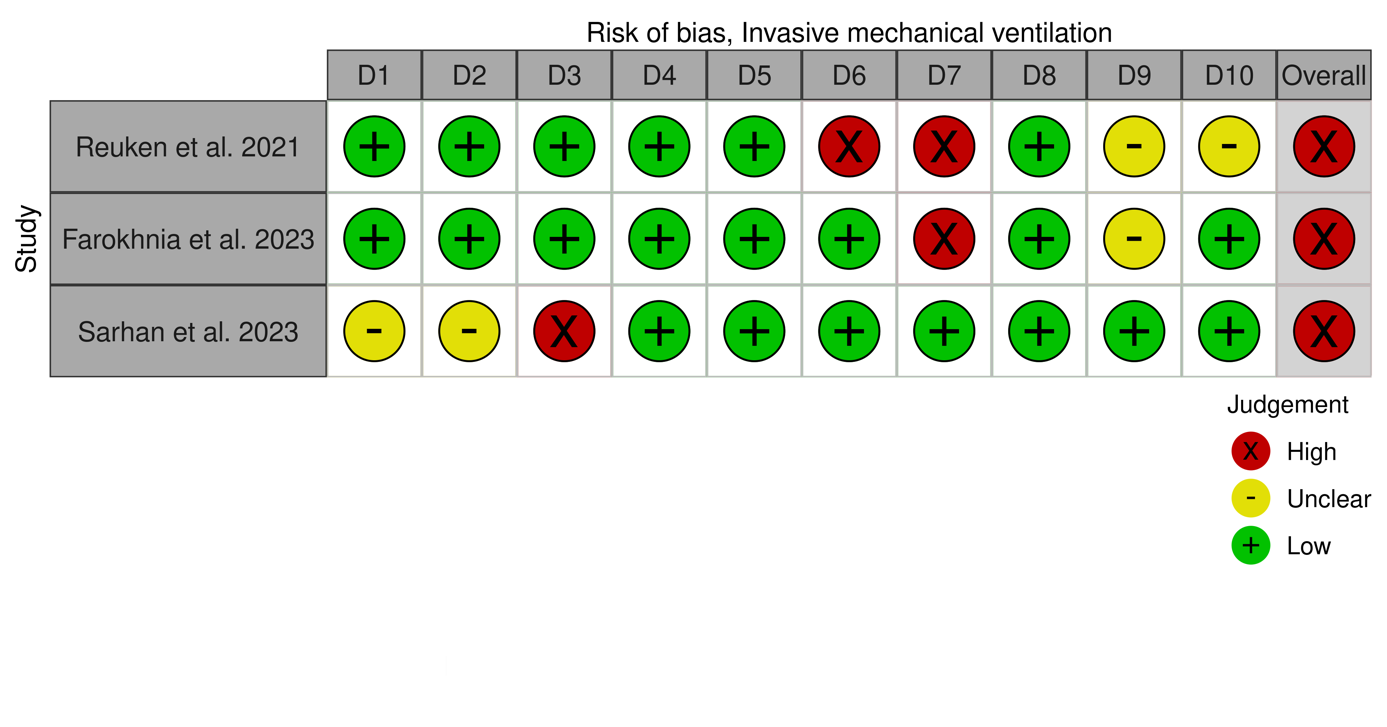


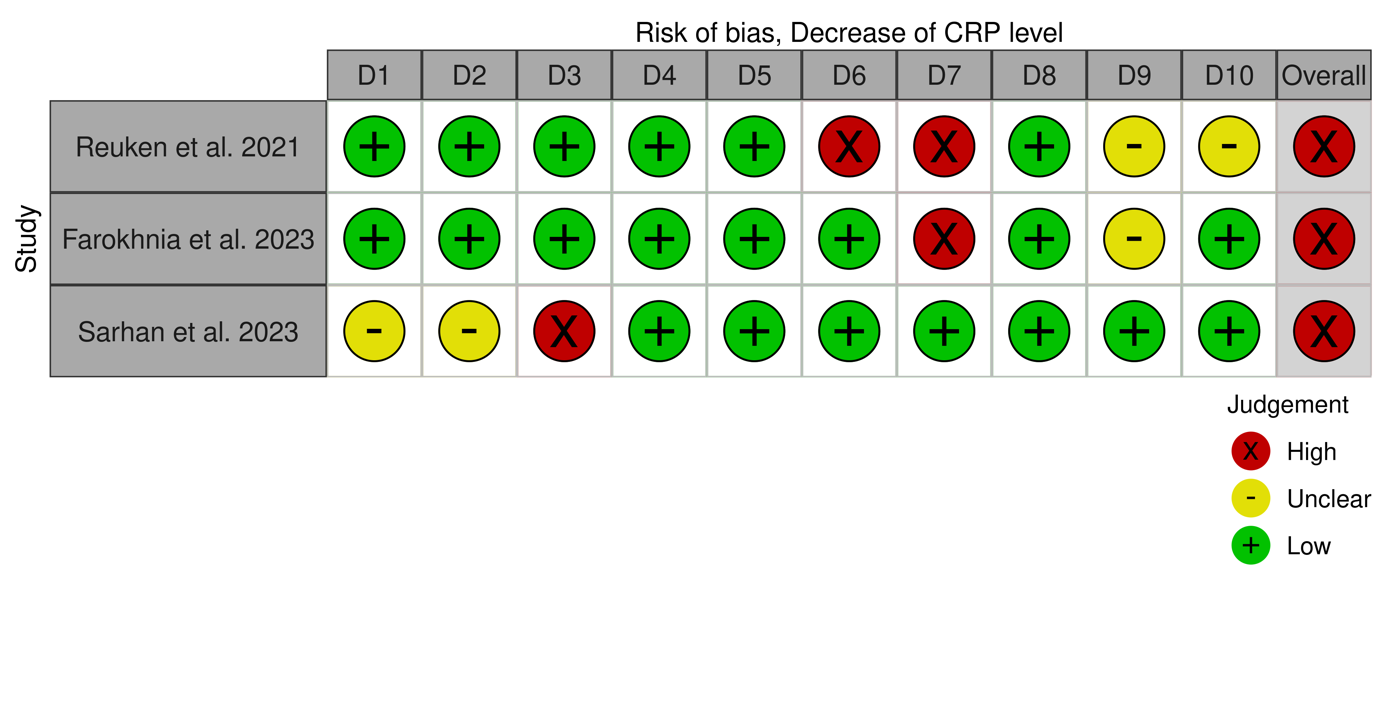


## Bibliography

1. Harrer M, Cuijpers P, Furukawa TA, Ebert DD. *Doing Meta-Analysis With R: A Hands-On Guide*. 1st ed. Chapman & Hall/CRC Press; 2021. https://www.routledge.com/Doing-Meta-Analysis-with-R-A-Hands-On-Guide/Harrer-Cuijpers-Furukawa-Ebert/p/book/9780367610074

2. Higgins JPT TJCJCMLTPMWV (editors). *Cochrane Handbook for Systematic Reviews of Interventions Version 6.4 (Updated August 2023). Cochrane, 2023. Available from Www.Training.Cochrane.Org/Handbook.*; 2023.

3. Luo D, Wan X, Liu J, Tong T. Optimally estimating the sample mean from the sample size, median, mid-range, and/or mid-quartile range. *Stat Methods Med Res*. 2018;27(6):1785-1805. doi:10.1177/0962280216669183

4. Wan X, Wang W, Liu J, Tong T. Estimating the sample mean and standard deviation from the sample size, median, range and/or interquartile range. *BMC Med Res Methodol*. 2014;14(1):135. doi:10.1186/1471-2288-14-135

5. Sterne JAC, Sutton AJ, Ioannidis JPA, et al. Recommendations for examining and interpreting funnel plot asymmetry in meta-analyses of randomised controlled trials. *BMJ*. 2011;343(jul22 1):d4002-d4002. doi:10.1136/bmj.d4002

6. Fakharian A, Barati S, Mirenayat M, et al. Evaluation of adalimumab effects in managing severe cases of COVID-19: A randomized controlled trial. *Int Immunopharmacol*. 2021;99(107961):107961. doi:10.1016/j.intimp.2021.107961

7. Fisher BA, Veenith T, Slade D, et al. Namilumab or infliximab compared with standard of care in hospitalised patients with COVID-19 (CATALYST): a randomised, multicentre, multi-arm, multistage, open-label, adaptive, phase 2, proof-of-concept trial. *Lancet Respir Med*. 2022;10(3):255-266. doi:10.1016/S2213-2600(21)00460-4

8. O’Halloran JA, Ko ER, Anstrom KJ, et al. Abatacept, Cenicriviroc, or Infliximab for Treatment of Adults Hospitalized With COVID-19 Pneumonia. *JAMA*. 2023;330(4):328. doi:10.1001/jama.2023.11043

9. Farokhnia M, Nakhaie M, Shafieipour S, et al. Assessment of the Effect of Sub-Cutaneous Adalimumab on Prognosis of COVID-19 Patients: a Non-Randomized Pilot Clinical Trial Study in Iran. *Clin Lab*. 2023;69(09/2023):1962-1968. doi:10.7754/Clin.Lab.2023.230343

10. Farrokhpour M, Rezaie N, Moradi N, et al. Infliximab and Intravenous Gammaglobulin in Hospitalized Severe COVID-19 Patients in Intensive Care Unit. *Arch Iran Med*. 2021;24(2):139-143. doi:10.34172/aim.2021.22

11. Reuken PA, Rüthrich MM, Hochhaus A, et al. The impact of specific cytokine directed treatment on severe COVID-19. *Leukemia*. 2021;35(12):3613-3615. doi:10.1038/s41375-021-01411-1

12. Sarhan NM, Warda AEA, Ibrahim HSG, Schaalan MF, Fathy SM. Evaluation of infliximab/tocilizumab versus tocilizumab among COVID-19 patients with cytokine storm syndrome. *Sci Rep*. 2023;13(1):6456. doi:10.1038/s41598-023-33484-6

13. Lin DY, Wang J, Anstrom KJ, et al. Efficacy of infliximab, abatacept, and cenicriviroc for the treatment of adults hospitalized with COVID-19 pneumonia. *International Journal of Infectious Diseases*. 2024;146:107168. doi:10.1016/j.ijid.2024.107168

14. La Rosée F, Bremer HC, Gehrke I, et al. The Janus kinase 1/2 inhibitor ruxolitinib in COVID-19 with severe systemic hyperinflammation. *Leukemia*. 2020;34(7):1805-1815. doi:10.1038/s41375-020-0891-0

15. Schünemann H, Brożek J, Guyatt G OA. *GRADE Handbook for Grading Quality of Evidence and Strength of Recommendations. Updated October 2013.*; 2013. https://gdt.gradepro.org/app/handbook/handbook.html

16. Sterne JAC, Savović J, Page MJ, et al. RoB 2: a revised tool for assessing risk of bias in randomised trials. *BMJ*. 2019;366:l4898. doi:10.1136/bmj.l4898

17. Moola S, Munn Z, Tufanaru C, Aromataris E, Sears K, Sfetcu, R Currie M, Qureshi R, Mattis P, Lisy K MPF. Checklist for Case Control Studies. *Joanna Briggs Institute Critical Appraisal tools*. Published online 2016:1-6.
